# Effects of Habitat Differences and Invasive Species Competition on Age and Growth of *Triplophysa strauchii*

**DOI:** 10.3390/ani15142128

**Published:** 2025-07-18

**Authors:** Ya-Han Meng, Wei-Zhen Gao, Yan Li, Lei Shi

**Affiliations:** 1 Xinjiang Key Laboratory for Ecological Adaptation and Evolution of Extreme Environment Organism, College of Life Sciences, Xinjiang Agricultural University, Urumqi 830052, China; m15201008090@163.com (Y.-H.M.); gao7222@yeah.net (W.-Z.G.); a18119152762@163.com (Y.L.); 2 College of Animal Sciences, Xinjiang Agricultural University, Urumqi 830052, China

**Keywords:** *Triplophysa strauchii*, lapillus, age identification, growth characteristics, invader competition

## Abstract

Accurate age determination is fundamental for investigating fish population dynamics and growth patterns. This study used the lapillus to determine age in *Triplophysa strauchii* populations from an oxbow lake and a stream. Growth patterns were evaluated using three models (the Von Bertalanffy, Gompertz, and Logistic models). The oxbow lake population showed faster growth and longer lifespan (6 years in Dacao Lake; 5 years in Liutiao Stream). Conversely, the stream population displayed a trend toward smaller size and younger age. Both populations exhibited higher Fulton’s condition factor in juveniles than in adults. The species exhibited a fast-growth type, with similar fitting results across models. These findings reflect subtle differentiation in life history strategies across habitats, likely related to environmental conditions and competitive pressure from invasive species. These insights into *T. strauchii* life history underscore the importance of further research to support conservation and sustainable management of this endemic species.

## Simple Summary

Fish growth traits constitute key adaptive attributes to environmental conditions, with growth variation representing the most direct and common response to habitat challenges. Distinct aquatic environments shape divergent life-history strategies and population characteristics in *Triplophysa strauchii*, whose geographically isolated populations experience significant habitat variation in factors including resource availability and interspecific competition. Through lapillus microstructure analysis of stream versus oxbow lake populations, we demonstrate that oxbow lake habitats—characterized by abundant food resources and reduced competition—support greater longevity and larger body sizes, whereas stream populations exposed to biological invasions exhibit reduced lifespan and body size miniaturization. This differential susceptibility to environmental fluctuations highlights conservation vulnerabilities in this endemic species. Population-level analyses further reveal substantial growth potential in the Dacao Lake, contrasting with prevalent miniaturization in the Liutiao River. Effective conservation thus requires preserving native habitats, preventing and controlling invasive species, and implementing long-term monitoring of fishery resource dynamics.

## 1. Introduction

Accurate determination of fish age is indispensable for elucidating population dynamics, assessing ecological adaptability, and forecasting population trends [1,2,3]. Calcified tissue analysis is a well-established method for evaluating population age structure [2,4]. Environmental fluctuations influence the deposition of calcium in fish hard tissues, creating annual rings [5]. These rings are used to deduce fish age [6]. For precise age determination, it is crucial to rely on high-precision structural materials [7]. Otoliths are formed of CaCO_3_ and protein [8,9] and grow continuously throughout a fish’s lifespan [10]. They exhibit non-cellular and metabolic inertness [11] and lack the capacity for reabsorption [12]. These characteristics make otoliths highly reliable for fish age determination [13,14,15]. Otoliths are categorized into lapilli, asteriscus, or sagittae [16]. Lapilli are relatively stable, and the annual rings are easy to read [17], thus making age identification more accurate.

The growth patterns of fish typically vary among species and can diverge within species along environmental gradients, such as latitude [18], diet [19], temperature [20], or population density [21]. Currently, three main methods are used in fish growth research: the direct method, age-based statistical analysis, and the back-calculation method [22]. Among these, the age-based statistical analysis involves compiling data on body length and weight across different age groups to characterize growth patterns. Fitting growth models using mathematical approaches facilitates comparative analyses [23]. The Von Bertalanffy growth function (VBGF), currently the most widely applied model, estimates fish growth by utilizing either directly measured or inferred age data.

The genus *Triplophysa* represents a highly diverse group within the family Cobitidae (order Cypriniformes) [24] and is distributed in most water areas of Xinjiang [25]. The ongoing tectonic uplift of the Tibetan Plateau and its adjacent mountain ranges has served as a primary driver for both the evolutionary origin and subsequent diversification of this genus [26,27]. Owing to its exceptional morphological differentiation and ecological adaptability, *Triplophysa* has garnered considerable attention from scholars across various domains, including taxonomy [28,29], genomics [30], evolutionary biology [26,31], and basic biological characteristics [32]. Among them, there are relatively few basic biological studies, such as the research on the growth and reproduction, and genetic diversity of *T. yarkandensis* [33], the classification and morphology of *T. stoliczkae* [34], and the age and growth patterns of *T. orientalis* [35] and *T. markehenensis* [36]. In recent years, *Triplophysa* has experienced a precipitous decline due to escalating human-induced disturbances and the invasion of alien species [37]. Therefore, it is imperative to focus on the study and conservation of these species.

The distribution area of *T. strauchii* in China is mainly in the Xinjiang Uygur Autonomous Region, and the species inhabits rivers along the northern slopes of the Tianshan Mountains [38]. Current research on this species has been limited to preliminary studies of its biological traits [38,39] and a description of its mitochondrial genome [40]. *T. strauchii* typically occupies small, cold, and pristine stream habitats characterized by oligotrophic conditions [41]. These habitats have low nutrient availability, limited primary productivity, and scarce food resources. Notably, Guo et al. [38] reported that populations in Chaiwopu Lake exhibited miniaturization and high parasitic infection rates due to competition from invasive fish species.

In this paper, the age structure and growth pattern of two geographically distinct populations of *T. strauchii* (stream-dwelling vs. oxbow lake-dwelling) were analyzed. The effects of divergent aquatic environments on fish growth were discussed. Specifically, the Liutiao Stream population faces a serious invasion of alien species, including benthic *Abbottina rivularis* and *Misgurnus anguillicaudatus,* which compete for the living space of *T. strauchii* [42,43]. In addition, *M. anguillicaudatus* demonstrates three invasive traits: (1) rapid environmental adaptation, (2) high fecundity, and (3) generalist benthic feeding behavior [44]. Research indicates that under equivalent feeding conditions, dominant species exhibit faster growth rates compared to subordinate species [45]. This indirectly reflects that such competition can directly impact the normal growth characteristics of *T. strauchii*. [46]. Conversely, no exotic species were detected in Dacao Lake. *Phoxinus grumi*, a sympatric native species, is a mid-upper layer, diurnal fish. Dietary analysis showed that *P. grumi* mainly feeds on zooplankton, and *T. strauchii* is polyphagous (unpublished data). Consequently, significant niche differentiation exists between *P. grumi* and *T. strauchii* across spatial, temporal, and trophic dimensions. This niche partitioning suggests *T. strauchii* experiences relatively reduced ecological competition within this lake ecosystem (Table A1) [47].

Driven by different survival pressures (habitat utilization, interspecific competition, etc.). It is predicted that the growth patterns of *T. strauchii* from both the oxbow lake and stream might be significantly different. The lake population, which experiences sufficient resources and less competition, is likely to exhibit better growth. Research on their growth pattern would be conducive to understanding the current status of wild resources and the population growth potential of this native species. It may also serve as a theoretical foundation for the conservation of *T. strauchii*.

## 2. Materials and Methods

### 2.1. Sample Collection

A total of 218 and 101 specimens of *T. strauchii* were collected during May 2024 from two distinct localities: 1. Dacao Lake (DL), Dabancheng District, Urumqi, Xinjiang Uygur Autonomous Region (88°24′ E, 43°21′ N; altitude 1169 m); 2. Liutiao Stream (LS), Balikun County, Hami, Xinjiang Uygur Autonomous Region (92°59′ E, 43°39′ N; altitude 1650 m). The fish were captured using cage nets (nets with a length of 5 m, a width of 3 m, and a mesh size of 4 mm). For detailed information on the catch composition, please refer to Table A1. DL is an oxbow lake formed in the middle section of the Baiyang River. It has a relatively small water surface and a slow flow rate, and it connects with the main river course. LS has a muddy and sandy bottom. The captured individual fish were anesthetized, fixed with 10% formaldehyde, then stored and brought back to the lab for further analysis. All experiments and animal handling were conducted according to research protocols approved by the Animal Welfare and Ethics Committee of Xinjiang Agricultural University.

### 2.2. Lapillus Processing and Age Determination

**Figure 1:**
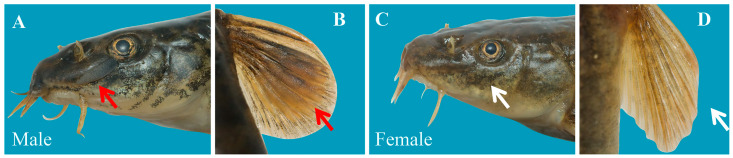
Comparison of female and male secondary sexual characteristics of *Triplophysa strauchii* in Dacao Lake. The arrow points to the prominent supra-opercular bone (**A**) and the thickened and enlarged fin rays (**B**) in males, while females do not have these features (**C**,**D**).

Specimens were numbered after fixation, and sex identification was conducted based on the species’ distinct secondary sexual characteristics. External morphological criteria included the presence of posterior or anterior processes on the lateral ethmoid bone (with cartilaginous buds) in males, which were covered by skin and projected below the anterior margin of the eyes (Figure 1A). In males, several outer pectoral fin rays were hardened and broadened, with dense, small spinules (villous nodules) on the dorsal surface (Figure 1B). Once developed, male secondary sexual characteristics persist throughout life. Females lacked these features (Figure 1C,D). Additionally, sexing was difficult in some small individuals, requiring dissection to observe gonad type and developmental stage. If both methods failed, the individual was classified as a juvenile [48].

Subsequently, with the left side as a reference, body length (BL, the horizontal distance from the tip of the snout to the base of the caudal fin) and total length (TL, the distance from the tip of the snout to the end of the caudal fin) were measured using an electronic digital caliper. The body weight was measured using an electronic balance [49]. The measurement data of the fish body length were recorded with an accuracy of 0.01 mm, and the weight measurements were recorded with an accuracy of 0.01 g.

The lapilli were fixed on glass slides and polished with 1500-grit sandpaper. They were moistened with a small amount of water until the central primordium and growth rings were clearly visible under an optical microscope. Then they were sealed on slides using neutral gum [50], photographed under an inverted BA210 Digital LED-Motic Microscopes (Motic, Xiamen, China), and archived for subsequent age determination [51].

**Figure 2:**
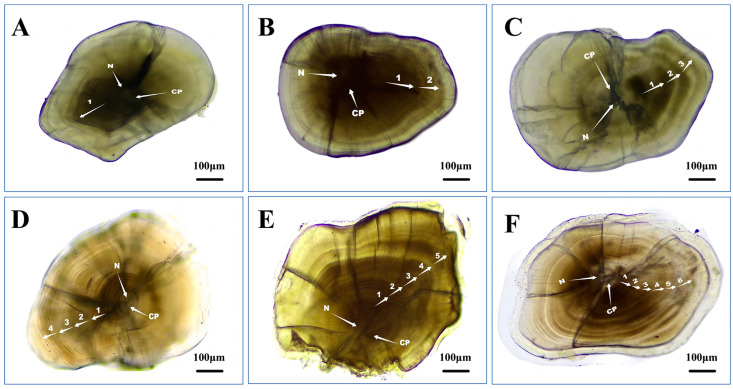
Lapillus of *Triplophysa strauchii* in Dacao Lake, showing different age groups (arrows show scale annuli). CP: central primordium; N: nucleus, 100×. (**A**): 1 year-old; (**B**): 2 year-old; (**C**): 3 year-old; (**D**): 4 year-old; (**E**): 5 year-old; (**F**): 6 year-old.

Yahan Meng and Yan Li conducted the initial reading of the otoliths (using double-blind reading). Weizhen Gao combined and evaluated the readings of the two experimental operators and re-read the inconsistent data, and his judgment was final (Figure 2).

In this study, 218 lapilli were extracted from *T. strauchii* in DL, and clear annual rings suitable for age determination were found in 195 lapilli. Moreover, 101 lapilli were extracted from *T. strauchii* in LS, and clear annual rings suitable for age determination were found in 74 lapilli. The inability of some individuals to have their otoliths’ annual rings read may be due to factors such as varying degrees of calcification in the otoliths. However, the age of the majority of individuals can be accurately determined from their otoliths. Based on a large amount of accurate body length and age data, we have drawn a body length–age frequency distribution chart. The results show that these data meet the assumption that “individuals of the same generation have similar body lengths, while those of different generations have different body lengths [15,52].” Therefore, we can match the body length range of some individuals whose age cannot be determined through otoliths with that of individuals of known ages to determine their age [53].

### 2.3. Data Analysis

#### 2.3.1. Length–Weight Relationship

The Keys formula can be used to perform regression analysis on the length–weight relationship [22]. The formula is $W={aL}^{b}$, where *W* is the body weight (g), *L* is the body length (mm), *a* is the condition factor, and *b* is the allometric growth factor. The Pauly *t*-test is used to determine if there is a significant difference between the *b* value and 3, which helps to identify the growth type of *T. strauchii*. The formula for the *t*-test is $t=\frac{SD(L)}{SD(W)}\times \frac{\left|b-3\right|}{\sqrt{1-r^{2}}}\times \sqrt{n-2}$, where *SD*(*L*) and *SD*(*W*) are the standard deviations of the body length and weight, respectively, *n* is the sample size, and *r*^2^ is the correlation coefficient from the length–weight equation [54].

#### 2.3.2. Fulton’s Condition Factor

The Fulton’s condition factor (*K*), also called Fulton’s coefficient, serves as a crucial biometric index for assessing fish’s nutritional status and body condition. It also helps evaluate their responses to environmental factors, providing valuable guidance for fisheries management. It was initially proposed by Fulton and expressed as a percentage, the formula is $K=(\frac{W}{L^{3}})\times 10^{5}$, where *W* is the body weight (g), and *L* is the body length (mm) [55].

#### 2.3.3. Growth Models

The growth pattern is one of the most crucial biological characteristics in the study of fish population dynamics [56,57]. In this study, we selected three commonly used growth models (Table 1). They were analyzed to compare the growth characteristics of *T. strauchii* in DL and LS, respectively. The equations were based on the premise that a linear correlation existed between the instantaneous growth rate and the logarithm of the body weight of the fish.

#### 2.3.4. Statistical Analysis

Morphological data were expressed as mean ± standard deviation (mean ± SD). The data were tested for normal distribution and homogeneity of variance. If the data conformed to both normal distribution and homogeneity of variance, one-way ANOVA was used to analyze differences between age groups in each basin. The LSD method was applied for multiple comparisons, and the results were labeled using the alphabetical method. For non-normally distributed data, a non-parametric test (e.g., Mann–Whitney U test for two groups) was used to analyze differences between groups. Statistical significance was set at *p* < 0.05. Analyses were performed using IBM SPSS 21.0, and nonlinear fitting of the growth curve was conducted with Origin 2024 software (Version 10.1).

## 3. Results

### 3.1. Body Length Distribution

**Figure A1:**
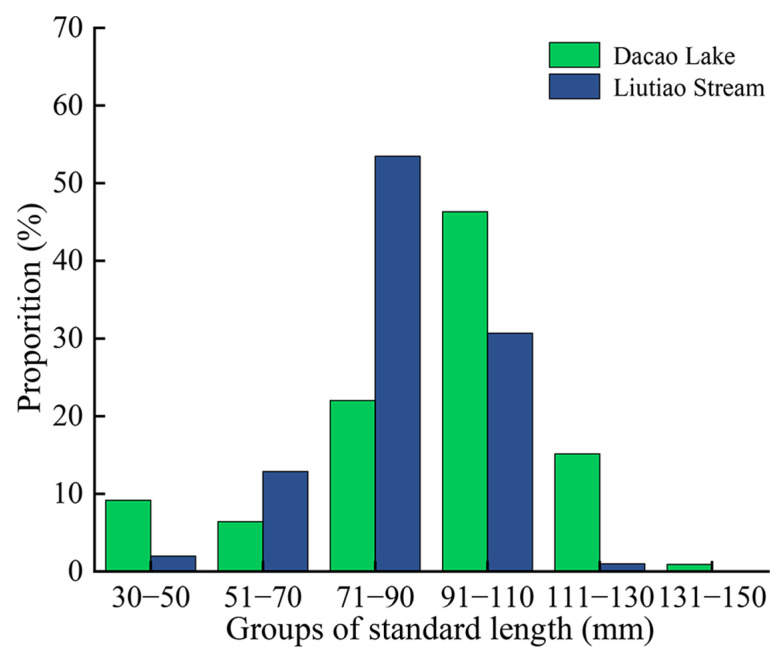
The distribution of body length of *Triplophysa strauchii*.

The body length of the specimens collected from DL ranged from 33.46 mm to 139.32 mm, with a mean (±SD) of 41.16 ± 12.72 mm. The dominant body length group ranged from 91 mm to 110 mm, comprising 90 individuals, accounting for 46.88% of the total. The second most frequent group was 71–90 mm, with 47 individuals, representing 24.48% of the population. In LS, the body length of the specimens ranged from 39.27 mm to 114.68 mm, with a mean (±SD) of 83.08 ± 12.92 mm. The dominant body length group ranged from 71 mm to 90 mm, comprising 54 individuals, and accounting for 53.47% of the total. The second most frequent group was 91–110 mm, with 31 individuals, representing 30.69% of the population (Figure A1).

### 3.2. Length–Weight Relationship

**Figure 3:**
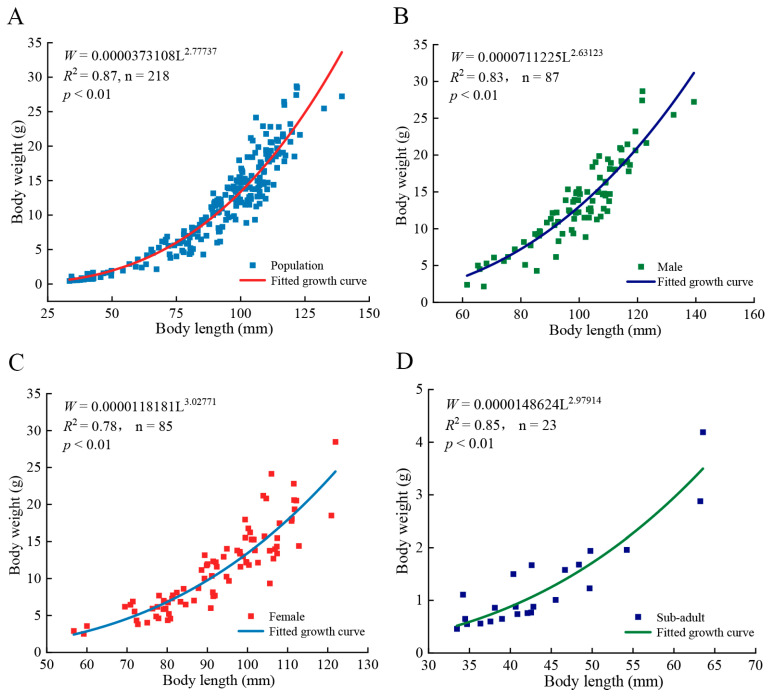
Length–weight relationships of *Triplophysa strauchii* in Dacao Lake. The relationship between body length and weight across the entire *Triplophysa strauchii* population (**A**), male (**B**), female (**C**), and juvenile (**D**) differentiation based on body length–weight relationships.

The body length of *T. strauchii* in DL was primarily distributed between 75 mm and 125 mm (Figure 3A). The results of the Pauly *t*-test, based on the corresponding *t* value and degrees of freedom (*t* = 29.68, *p* < 0.05), indicate that the population of *T. strauchii* in DL exhibited a negative allometric growth, implying the growth rate in length was faster than in weight. The *b* for males (Figure 3B) and sub-adult (Figure 3D) were 2.63 and 2.98, respectively, both significantly lower than 3 (*t*_♂_ = 22.40, *t* = 2.20, *p* < 0.05), indicating a negative allometric growth. In contrast, the *b* for females (Figure 3C) was 3.03, showed no significant difference from 3 (*t*_♀_ = 1.40, *p* > 0.05), suggesting an isometric growth.

**Figure 4:**
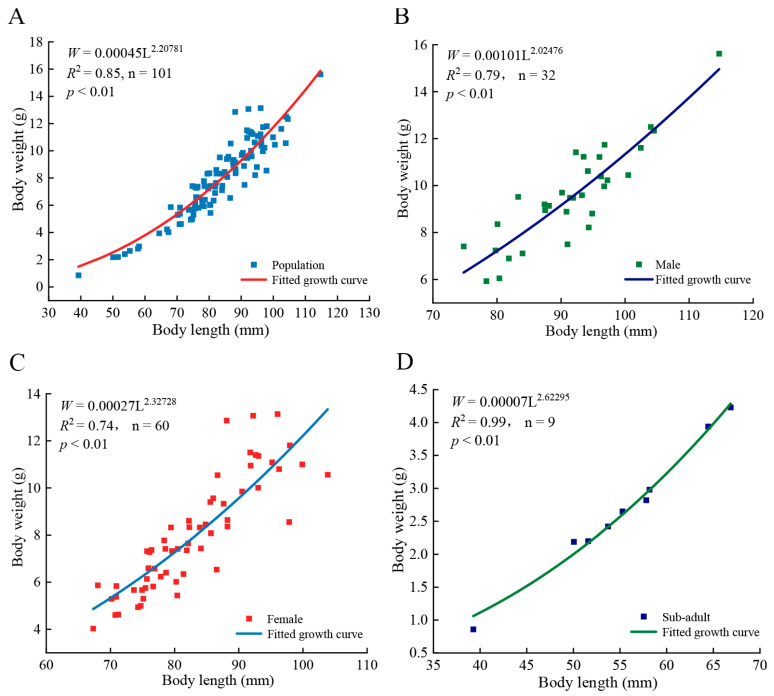
Length–weight relationships of *Triplophysa strauchii* in Liutiao Stream. The relationship between body length and weight across the entire *Triplophysa strauchii* population (**A**), male (**B**), female (**C**), and juvenile (**D**) differentiation based on body length–weight relationships.

The body length of *T. strauchii* in LS was primarily distributed between 75 mm and 95 mm (Figure 4A). The *b* was 2.21 < 3 (*t* = 18.03, *p* < 0.05), indicating that the population exhibited a negative allometric growth. The *b* for males (Figure 4B) and females (Figure 4C) were 2.02 and 2.33, respectively, both significantly lower than 3 (*t*_♂_ = 5.14, *t*_♀_ = 3.79, *p* < 0.05), indicating a negative allometric growth. In contrast, the *b* for sub-adult (Figure 4D) was 2.62 (*t* = 0.04, *p* > 0.05), suggesting an isometric growth.

### 3.3. Age Structure

**Figure 5:**
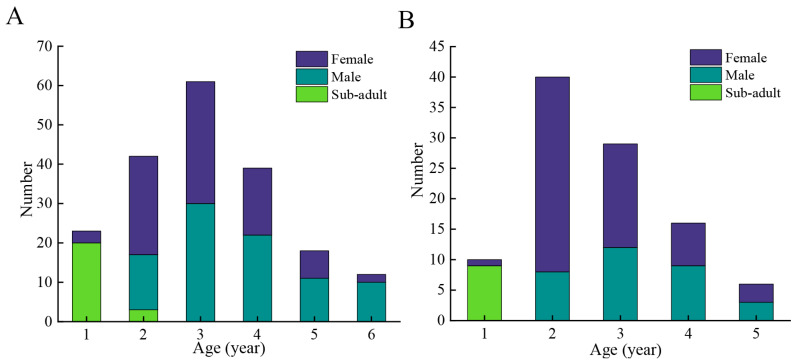
Age distribution of *Triplophysa strauchii* in Dacao Lake (**A**) or Liutiao Stream (**B**).

The age of the specimens in DL ranged from 1 to 6 years. The dominant age was 3 years. In LS, the age of *T. strauchii* ranged from 1 to 5 years. The dominant age was 2 years (Figure 5). One-way ANOVA revealed significant differences in body length between populations in different basins. The results revealed that male *T. strauchii* aged 1 and 2 in LS were significantly larger than their counterparts in DL (*p* = 0.001, 0.038 < 0.05). Conversely, males aged 3 or 4 from DL were significantly larger than those from LS (*p* = 0.021, 0.000 < 0.05). Female *T. strauchii* aged 3, 4, or 5 from DL exhibited significantly greater body length compared to those from LS (*p* = 0.000, 0.000, 0.000 < 0.05). Within the same basin, sexual dimorphism was observed in 2-year and 3-year individuals from LS, where males were significantly larger than females (*p* = 0.008, 0.036 < 0.05) (Table A2).

### 3.4. Fulton’s Condition Factor

**Figure 6:**
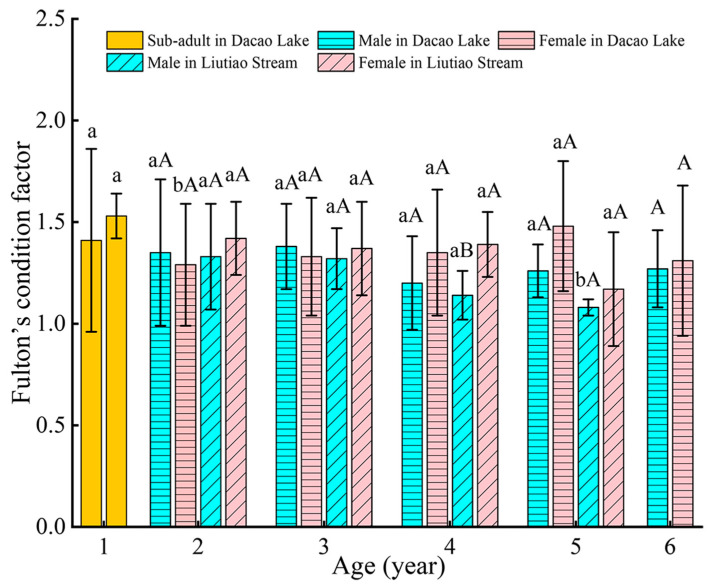
The condition factor of *Triplophysa strauchii* across different age groups. Significance difference was deemed at *p* < 0.05. Differences between the two geographic populations within the same sex were indicated by lowercase letters, while differences between sexes within each age group were denoted by uppercase letters. The same letter indicates no significant difference, whereas different letters represent a statistically significant difference.

Both DL and LS populations of *T. strauchii* exhibited higher condition factors in 1-year juveniles compared to adults. Female condition factors of 2-year, *T. strauchii* in LS were significantly greater than those of 2-year males in DL (*p* = 0.004 < 0.05). The male condition factor of 5-year individuals in DL was significantly higher than that of 5-year males in LS (*p* = 0.022 < 0.05). Within the population, comparisons showed female condition factors of 4 years in LS displaying significantly higher than males (*p* = 0.048 < 0.05) (Figure 6).

### 3.5. Growth Models

**Figure 7:**
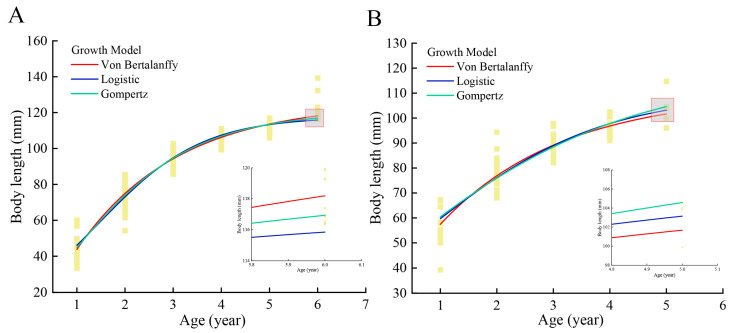
The different growth models fitted to *Triplophysa strauchii* in Dacao Lake (**A**) or Liutiao Stream (**B**).

Body length and age data were fitted using nonlinear regression with Von Bertalanffy, Logistic, and Gompertz growth models (Table A3 and Figure 7).

The VBGM predicted the largest asymptotic body length *L_∞_* (125.667 mm), while the Logistic model yielded the smallest estimate (117.537 mm) in DL. Conversely, the Logistic model produced the highest *L_∞_* (115.647 mm), with the VBGM showing the lowest value (102.782 mm) in LS. The specific fitting results of each growth equation, along with the coefficient of determination (*R*^2^) and residual sum of squares (RSS), were presented in Table A3. The fitting results of the three growth models for the species were similar and effectively described the species’ growth pattern. The growth rate in body length was maximized at 1 year, and it slowed down with increasing age, approaching the asymptotic body length (Figure 7A,B).

## 4. Discussion

### 4.1. Growth Characteristics

The growth characteristics of fish are the results of the interaction between external (environmental) and internal (biological) factors, primarily reflected in body length and weight relationships. Even within the same species, population growth characteristics can diverge significantly under differing environmental conditions [2,61]. Nutritional conditions, habitat states, fishing pressure, and interspecific competition also affect the population characteristics [62,63,64]. Therefore, the growth parameter *b* always changes [65]. When *b* is close to 3, it typically suggests an isometric growth. When *b* is close to 3, this typically indicates isometric growth; when *b* is less than 3, it indicates negative allometric growth; when *b* is greater than 3, it indicates positive allometric growth [66]. In this study, the *b* value for *T. strauchii* in either DL (*b* = 2.78) or LS (*b* = 2.21) was significantly lower than 3 (*p* < 0.05), indicating negative allometric growth.

The DL was characterized by relatively sluggish water flow, a predominantly muddy substrate, and abundant aquatic weeds, thereby offering a plentiful food source. These conditions were likely conducive to the rapid growth of *T. strauchii*. In contrast, the LS predominantly had a gravel substrate, with fast-flowing water and barely any aquatic weeds. The food resources were scarce, and the species also faced direct competition from invasive species occupying the same ecological niche. Consequently, the growth rate of *T. strauchii* in the LS was lower, and their body size was smaller. It can be inferred that the same species can exhibit different growth characteristics in response to various habitat types and resource conditions [62,63].

### 4.2. Age Structure

The population of *T. strauchii* exhibited a relatively low average age and a straightforward age structure. Currently, *T. strauchii* in DL aged 1 to 2 years accounted for 29.36%, while individuals aged 3 to 6 years comprised 70.64%. This indicates that *T. strauchii* in this area faces lower environmental pressure, with a low proportion of young individuals. *T. strauchii* in LS aged 1 to 2 years accounted for 75.68%, while individuals aged 4 to 5 years comprised 24.32%, demonstrating a distinct younger age structure. The population age structure of *T. strauchii* in DL or LS was primarily composed of juveniles and young adults. These findings were similar to observations of *T. strauchii* in Sailimu Lake [39]. In contrast, individuals from Chaiwopu Lake showed considerable signs of parasitic infections [38]. These results are consistent with the growth patterns of *T. yarkandensis*, whose population exhibits faster growth in better habitats [13]. Field surveys revealed significant invasive species presence in the watershed of LS, where benthic competitors, including *M. anguillicaudatus* and *A. rivularis*, were observed to overlap ecologically with the native *T. strauchii*. This interspecific competition likely constrained habitat availability and food resources for *T. strauchii* [42,43]. As a consequence, the dominance of younger individuals and the smaller body size of *T. strauchii* in this water area indicated that the population is facing severe ecological pressure.

The growth inflection point was not only closely related to sexual maturity and aging, but also related to water temperature changes and nutritional conditions [67]. In this study, the growth inflection point for *T. strauchii* in DL and LS was 2.32 and 2.07 years, respectively, indicating that the majority of the individuals had surpassed the rapid growth and exhibited a relatively stable state [13]. In DL, the growth inflection points for males and females were 3.32 and 2.48, respectively. Their growth was obvious in the later stage, with delayed sexual maturity and minimal sexual dimorphism. Because females require continuous rapid growth to meet reproductive demands, whereas males show less observable growth in response to the development of sexual glands [68]. The LS population of *T. strauchii* exhibited more prominent male-bias sexual size dimorphism, with males significantly exceeding females in body length at 2 or 3 years (*p* < 0.05). The earlier growth inflection points (*t_IP_*_♀_ = 1.57; *t_IP_*_♂_ = 1.81) showed that the sexual maturity of fish in this population was early.

### 4.3. Fulton’s Condition Factor

The condition factor (*K*) serves as a crucial biological indicator in fish studies [55]. This metric is influenced by both environmental factors and intrinsic physiological characteristics and is correlated with gonadal development [69]. In this study, both the DL and LS populations of *T. strauchii* displayed higher condition factors in 1-year-old individuals. This pattern likely reflected substantial energy reserves during early development stages, which facilitated survival and early growth [70]. Comparative analysis revealed that females generally exhibited higher condition factors than males in both watersheds. Therefore, we hypothesized that enhanced female condition might promote gonadal growth and development, thereby increasing reproductive investment and offspring output to improve population recruitment.

### 4.4. Growth Models

VBGM is widely regarded as a general model suitable for fitting growth patterns in most fish species [23], though it performs poorly in simulating the early growth stages of juveniles. The Gompertz model is often preferred for modeling juvenile [71] or female growth patterns [72]. The Logistic model is better fitted for males [73,74]. However, this study found consistent performance among these three models, suggesting comparable suitability for fitting the growth pattern of *T. strauchii*. Therefore, the conventional VBGM was selected to compare the growth pattern of *T. strauchii* across different watersheds. The growth coefficient *k* reflects the growth rate of fish. A *k* value between 0.05 and 0.10 indicates a slow growth; a *k* value between 0.10 and 0.20 suggests a moderate growth; and a *k* value between 0.20 and 0.50 signifies a rapid growth [75]. The *k* values for *T. strauchii* in DL and LS were 0.451 and 0.335, respectively, indicating a fast-growing species. In the case of limited resources or increased environmental pressure (fishing, biological invasion, etc.). *T. strauchii* tended to allocate energy preferentially to sexual maturity (reproduction) rather than growth, resulting in early sexual maturity and reduced maximum body length (smaller size). These results are consistent with predictions of the energy trade-off hypothesis [76].

Overall, by comparing the age and growth characteristics of different geographical populations of *T. strauchii*, this study reveals that environmental differences and ecological competitive pressure significantly affect the growth of this fish. However, this experiment only focused on the growth of *T. strauchii* in a single season (May) and did not consider the influence of seasonal changes on growth, which may result in an incomplete generalization of the population’s overall growth patterns. In the future, we will continue to monitor the population dynamics of *T. strauchii*, adopt a longer time scale, expand to a wider range of geographical populations, and conduct controlled experiments to reveal how its growth characteristics change under different ecological competitive pressures, thereby offering more scientific support for the conservation of native fish species.

## 5. Conclusions

Fish normally develop unique life history strategies to adapt to specific aquatic environments, resulting in population-specific age structure and growth patterns. Our findings demonstrated that *T. strauchii* exhibited an allometric growth pattern across different habitats, indicating a fast-growing type. The population of *T. strauchii* in DL aged from 1 to 6 years. The dominant age group was 3-year-olds, with a growth inflection point at 2.32 years. *T. strauchii* in DL showed no signs of smaller size, younger age, and attained a larger asymptotic body length. In contrast, the population in LS exhibited an age range of 1–5 years, with a predominance of 2-year-old individuals and a growth inflection point at 2.07 years, demonstrating a distinct younger age structure and smaller size. The DL population experienced less ecological pressure and maintained a higher growth potential. In LS, the miniaturization and younger age of fish were more frequent, which might be caused by the deterioration of the water environment, the bait competition with alien species, or the limited nutritional resources. Therefore, the growth and population characteristics of *T. strauchii* are directly related to environmental conditions. Consequently, it is imperative to strengthen the conservation of the aquatic environment, safeguard against the invasion of alien species, maintain continuous surveillance of the fishery resources in the area, and implement scientifically informed conservation strategies.

## Figures and Tables

**Table 1:** The most commonly evaluated growth models.

Growth Model	Equation	Parameter Description	Reference
Von Bertalanffy	$L_{t}=L_{\infty }[1-e^{-k(t-t_{0})}]$	*L*_(*t*)_ = standard length at age *t* *L_∞_* = theoretical asymptotic length *t*_0_ = age at zero length *k* = growth parameter	[58]
Logistic	$t_{IP}=\frac{lnb}{k}+t_{0}$	*t_IP_* = growth inflection point	[59]
Gompertz	$L_{t}=L_{\infty }[e^{-e^{-G(t-t_{IP})}}]$ $G=ln(\frac{L_{\infty }}{L_{0}})$	*G* = instantaneous growth rate coefficient at age *t_IP_* *L*_0_ = the length at *t*_0_	[60]

## Data Availability

The dataset is available upon request from the authors.

## Appendix A

**Table A1:** Fishing catch statistics table.

Sampling Site (Time)	Species	Number	Proportion of Catch	Type	Feeding Habits	Activity Rhythm	Habitat Water Layer	References
Dacao Lake (27 April 2024)	*Triplophysa strauchii*	218	57.80%	Native	Omnivory	Nocturnal	Demersal	Unpublished data; [48]
	*Phoxinus grumi*	92	42.20%	Native	Omnivory (zooplankton)	Diurnal	Pelagic	Unpublished data
Liutiao Stream (19 May 2024)	*Triplophysa strauchii*	101	56.11%	Native	Omnivory	Nocturnal	Demersal	Unpublished data; [48]
	*Misgurnus anguillicaudatus*	55	30.56%	Alien	Omnivory	Nocturnal	Demersal	[43]
	*Abbottina rivularis*	24	13.33%	Alien	Omnivory	Diurnal	Demersal	[42]

**Table A2:** Body length of *Triplophysa strauchii* in different age groups (mm).

Age	Sex	Dacao Lake		Liutiao Stream		*p*-Value
		Mean ± SD	Range	Mean ± SD	Range	
1	Sub-adult	43.29–7.75 ^b^ (n = 23)	33.46–59.99	55.24 ± 8.16 ^a^ (n = 9)	39.27–66.86	0.001
2	Male	74.48 ± 8.06 ^bA^ (n = 14)	61.54–85.45	81.69 ± 5.75 ^aA^ (n = 8)	74.80–94.32	0.038
	Female	77.88 ± 4.35 ^aA^ (n = 25)	69.46–84.59	76.62 ± 4.28 ^aB^ (n = 32)	68.03–87.64	0.279
3	Male	94.34 ± 4.97 ^aA^ (n = 30)	85.72–102.76	90.55 ± 3.54 ^bA^ (n = 12)	83.31–96.74	0.021
	Female	94.35 ± 4.50 ^aA^ (n = 31)	86.67–101.84	87.28 ± 4.18 ^bB^ (n = 17)	82.10–97.85	0.000
4	Male	105.90 ± 2.76 ^aA^ (n = 22)	100.25–109.83	96.58 ± 3.36 ^bA^ (n = 9)	91.01–102.45	0.000
	Female	104.81 ± 3.23 ^aA^ (n = 17)	99.39–110.97	94.16 ± 2.34 ^bA^ (n = 7)	91.77–97.95	0.000
5	Male	112.55 ± 2.50 ^aA^ (n = 11)	109.09–116.96	107.73 ± 6.02 ^aA^ (n = 3)	104.01–114.68	0.298
	Female	110.96 ± 2.28 ^aA^ (n = 7)	105.95–112.79	99.92 ± 3.91 ^bA^ (n = 3)	96.03–103.85	0.000
6	Male	122.68 ± 7.46 ^A^ (n = 10)	116.43–139.32			
	Female	121.40 ± 0.72 ^A^ (n = 2)	120.89–121.91			

Notes: Significance difference was deemed at *p* < 0.05. Differences between the two different geographic populations within the same sex (within the same row) were indicated by lowercase letters, while differences between sexes within each age group (within the same column) were denoted by uppercase letters. The same letter indicates no significant difference, whereas different letters represent a statistically significant difference.

**Table A3:** Growth function of *Triplophysa strauchii* after fitting.

Model	Dacao Lake	*R* ^2^	RSS	Liutiao Stream	*R* ^2^	RSS
Von Bertalanffy	$L_{t}=125.667[1-e^{-0.451(t-0.059)}]$	0.9393	6.346	$L_{t}=105.307[1-e^{-0.335(t+1.241)}]$	0.9982	2.9738
Logistic	${L_{t}=117.537\left[1+e^{-0.433(t-2.32}\right]}^{-1}$	0.9309	7.234	${L_{t}=115.647\left[1+e^{-0.234(t-2.07}\right]}^{-1}$	0.8352	2.7506
Gompertz	$L_{t}=120.389[e^{{-e}^{-0.705(t-2.32)}}]$	0.9360	6.953	$L_{t}=102.782[e^{{-e}^{-0.715(t-2.07)}}]$	0.8454	5.9596
